# Piezosurgery-assisted protocol for impacted mandibular third molar removal with 3D facial swelling analysis: a randomized split-mouth clinical trial on post-surgical sequelae

**DOI:** 10.1007/s00784-026-06982-w

**Published:** 2026-06-18

**Authors:** Alessandro Antonelli, Giulia Brunello, Marianna Salviati, Selene Barone, Francesco Bennardo, Jason Motta Jones, Amerigo Giudice

**Affiliations:** 1 School of Dentistry, Department of Health Sciences, University of Catanzaro, Magna GraeciaViale Europa, 88100 Catanzaro, Italy; 2 Department of Oral Surgery, University of Düsseldorf, 40225 Düsseldorf, Germany; 3 Department of Orthodontics and Dentofacial Orthopaedics, Charité - Universitätsmedizin Berlin, corporate member of Freie Universität Berlin and Humboldt-Universität Zu Berlin, 14197 Berlin, Germany; 4 Humanitas University, Pieve Emanuele, 20072 Milan, Italy; 5 Humanitas Dental Center, Humanitas Research Hospital, 20089 Rozzano, Milano Italy; 6 A.O.U. Renato Dulbecco, Academic Hospital of Magna Graecia University of Catanzaro, 88100 Catanzaro, Italy

**Keywords:** Wisdom tooth, Piezoelectric surgery, Ultrasonic surgery, Edema, Pain, 3D facial scan

## Abstract

**Objectives:**

This split-mouth randomized controlled trial aimed to compare a novel piezosurgery protocol versus a conventional rotary technique for the extraction of mesio-angulated and horizontally impacted mandibular third molars (MA-HM3Ms), evaluating postoperative inflammatory sequelae and patient-centered outcomes.

**Materials and methods:**

Forty-seven healthy adult patients underwent bilateral MA-HM3M extraction with piezosurgery protocol (test group, TG) and, contralaterally, with conventional rotary instruments and manual elevators (control group, CG). Facial swelling was objectively quantified using three-dimensional facial scans at 2 and 7 days postoperatively. Secondary outcomes included postoperative pain, maximum mouth opening, bleeding, surgical time, analgesic intake, complications, and patient-reported outcomes assessed using the PoSSe scale. Statistical analysis was performed using paired comparisons and regression models (α = 0.05).

**Results:**

Ninety-four extractions were analyzed. After 2 days, lower facial swelling was significantly reduced in TG compared with CG (*p* = 0.01), together with less pronounced trismus (*p* = 0.016). Pain was consistently lower in the TG at all timepoints, while postoperative bleeding and analgesic intake were significantly higher in the CG (*p* < 0.05). Surgical time was longer in TG than in CG (*p* < 0.001). The piezosurgery protocol significantly reduced interference with daily activities and resulted in higher patient satisfaction. Regression analyses showed a direct association between swelling and PoSSe scale in both groups. No permanent neurological complications were observed; alveolitis occurred in 3 CG and 2 TG sites.

**Conclusions:**

This split-mouth randomized controlled trial suggests that piezosurgery for MA-HM3M extraction effectively reduces early postoperative inflammatory sequelae, improving patient-centered outcomes and quality of life, despite longer operative times.

**Clinical relevance:**

The proposed piezoelectric protocol could represent a minimally invasive alternative to conventional rotary techniques, particularly in complex MA-HM3M extractions, enhancing patient comfort and quality of life during the most critical postoperative period while supporting a more controlled and refined surgical approach.

**Registry: **ClinicalTrials.gov, TRN: NCT06212232, Registration date: 25 January 2024.

## Introduction

The mandibular third molar (M3M) is the most frequently impacted tooth in the human dentition [1]. Although prevalence rates vary across populations and geographic regions, some studies report overall impaction rates as high as 70% [2, 3]. Among various angulations and positions, mesio-angulated and horizontally impacted M3M (MA-HM3M) represents the most common clinical presentations encountered by practitioners in daily surgical practice [4, 5]. Several studies evaluating the developmental stage and eruption status of lower third molars have consistently shown that this specific pattern—MA-HM3M—can account for up to 80% of all M3M impactions, confirming its relevance and frequency in clinical settings [5, 6].

MA-HM3Ms can give rise to several clinical implications. When impacted or partially erupted, these teeth often create plaque-retentive areas on the distal surface of the adjacent second molars [7, 8]. This anatomical condition predisposes patients to pericoronitis, distal caries and periodontal defects affecting the second molar, as well as odontogenic abscesses and odontogenic cysts [9–12].

For these reasons, the extraction of impacted third molars is a widespread practice in oral and maxillofacial surgery. Although the complication rate is generally low and transient, this surgical procedure is associated to notable postoperative morbidities [13]. The removal of the impacted M3Ms often induces considerable surgical trauma to the surrounding soft tissues and bone structures, leading to a significant inflammatory reaction [14]. Postoperative inflammatory sequelae may result in symptoms of varying severity, such as pain, facial swelling, and limited mouth opening [15]. While these symptoms are relatively common, the surgical trauma can also lead to additional postoperative complications, including transient sensory disturbances in the chin and tongue regions, as well as alveolitis [16–18]. While most of these complications are temporary, they can have a great impact on the patient's psychological well-being, appearance, and overall quality of life [19]. To minimize these adverse effects and enhance the patient's recovery after surgery, clinicians have introduced several protocols, including the administration of perioperative non-steroid anti-inflammatory (NSAIDs) drugs or corticosteroids, the use of postoperative wound drains, various flap designs, autologous platelet concentrates use or low-dose laser therapy to promote wound healing [20–23]. Moreover, another crucial factor affecting postoperative outcomes is the osteotomy technique employed for the removal of the M3Ms. Although rotary handpiece instruments are commonly used for the removal of impacted teeth, some studies have reported irregular bone surfaces and heat generation at the margins, which could potentially affect the healing process [24, 25]. An alternative to rotary instruments is piezosurgery, which provides a more precise and controlled osteotomy by generating ultrasonic frequencies (24–29 kHz) with micro-vibrations, enabling selective bone cutting while minimizing the risk of injury to adjacent soft tissues, nerves, and vessels [26, 27]. Furthermore, the cavitation effect enhances hemostasis and intraoperative visibility, while proper piezoelectric osteotomy has been shown to reduce cellular stress and promote faster, more effective bone healing compared with rotary surgery [28, 29].

While many studies and systematic reviews have compared piezosurgery to the traditional rotary approach for M3M extraction, a new protocol has recently been introduced that employs piezoelectric instruments not only for pericoronal osteotomy but also for tooth luxation and extraction maneuvers [30, 31]. Fontanella et al. demonstrated that this approach reduces operative time and minimizes tissue trauma during third molar extraction procedures [31]. Although the authors presented interesting results from a large parallel-group study, the heterogeneity third molar types included limits the possibility to draw definitive conclusions regarding the comparative performance of the two surgical approaches [31].

The aim of this study was to assess and compare the clinical efficacy of piezosurgery, utilized for flap elevation, osteotomy and tooth luxation, against the traditional technique for M3M extraction in a split-mouth randomized clinical trial. Facial swelling was considered the primary outcome and was evaluated using 3D facial scanning. Secondary outcomes were postsurgical pain, maximum mouth opening, time of surgery, postoperative bleeding and patient’s quality of life.

## Material and methods

This study is reported according to the CONSORT statement and its extension for within-person randomized trials [32].

### Study design

All the procedures were conducted in accordance with the Declaration of Helsinki. This study was approved by the regional Ethical Review Board of Central Calabria (reference for Magna Graecia University of Catanzaro—No. 123/2023). The study was designed as a single-blind, split-mouth, randomized clinical trial and was registered at www.clinicaltrials.gov (NCT06212232) on January 25, 2024. The predictor variable in this study was the surgical protocol employed for mandibular third molar removal, which consisted of either a piezoelectric or a traditional technique.

### Study sample

Participants were recruited at the Dental Unit of “AOU—*R. Dulbecco*” of Magna Graecia University of Catanzaro, Italy. The inclusion criteria of the study were: (i) patients aged between 18 and 32 years in good general health who required the surgical extraction of both mandibular third molars (M3Ms); (ii) presence of both M3M in a mesio-angulated or horizontally impacted position (MA-HM3M) according to Winter classification [33]; complete root formation, i.e. Shumaker *Stage H* [34].

The exclusion criteria were as follows: (i) acute infection in any of the teeth scheduled for extraction; (ii) chronic liver disease, chronic kidney disease, diabetes, immune system dysfunction, or hematological disorders; (iii) pregnancy or breastfeeding; (iv) history of antiresorptive drug therapy; (v) and the use of NSAIDs or corticosteroids within the four weeks prior to surgery.

All participants received detailed information regarding the risks and potential benefits of the procedure and provided written informed consent prior to enrolment. Additionally, all patients signed consent forms authorizing the use of their data and/or photographs for scientific purposes.

### Randomization and blinding

The randomization process was performed using a computer-generated random shortlist. Each surgical protocol, piezoelectric surgery protocol, Test Group (TG) and traditional surgery protocol, Control Group (CG), was randomly assigned to a specific M3M (left or right). Identical, opaque envelopes containing the different possible combinations were opened prior to the first extraction, determining the allocation to the test or control group.

Randomization and allocation procedures were performed by an independent operator (S.B.) not involved in the surgeries and in the registration of the postoperative parameters. The oral surgeon (A.A.) was not blinded due to the experimental procedures. All postoperative parameters were recorded by the same blinded operator (M.S.) not involved in the randomization and surgical procedures.

### Surgical protocols

**Fig. 1:**
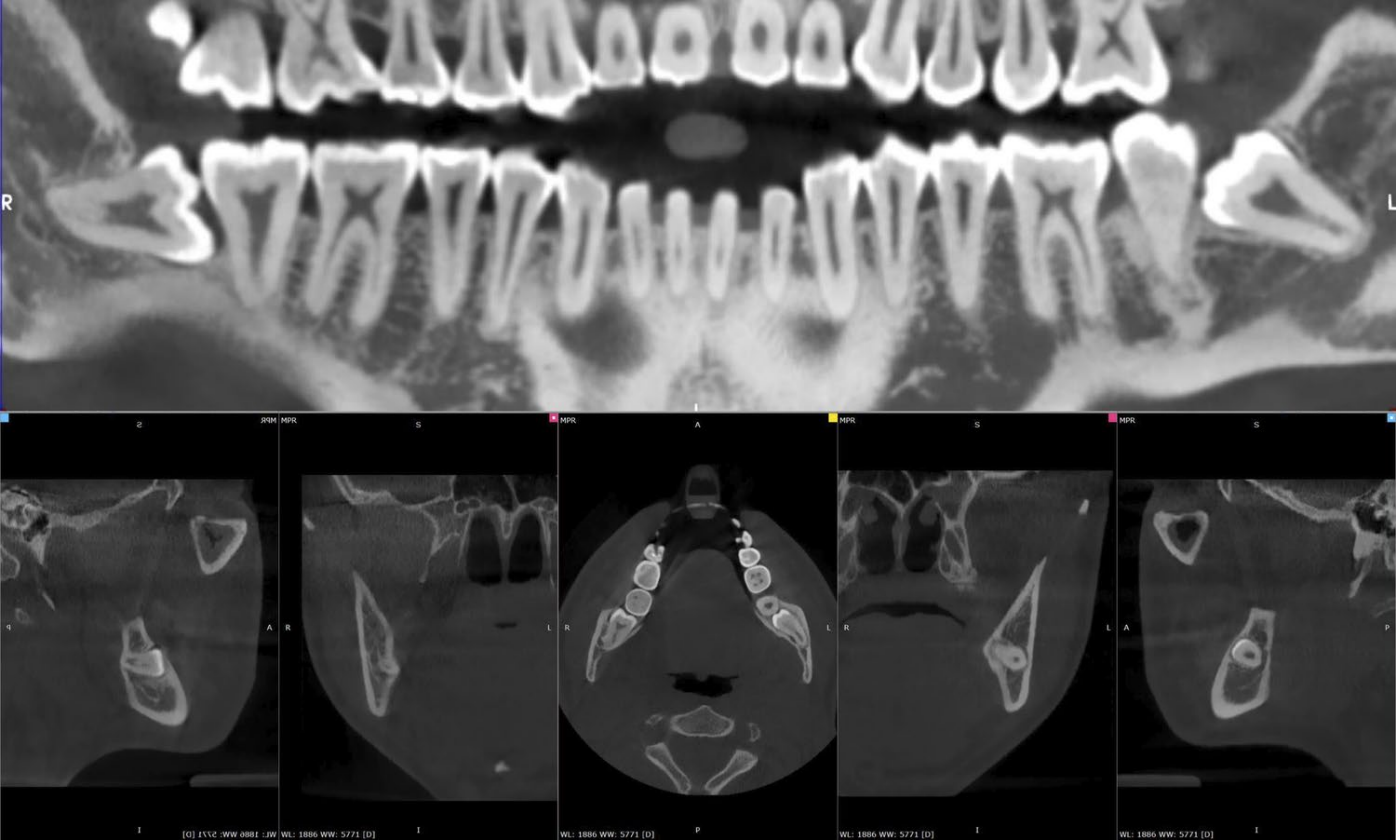
Representative preoperative CBCT assessment of MA-HM3Ms. Multiplanar reconstructions (axial, sagittal, and coronal views) depict tooth angulation, depth of impaction, root morphology, and proximity to adjacent anatomical structures, including the mandibular canal and second molar

All patients enrolled in this study underwent a preoperative cone-beam computed tomography (CBCT; X-Mind® Trium, Acteon®, Mérignac, France) to assess the position and morphological characteristics of the MA-HM3Ms. DICOM datasets were analyzed using dedicated imaging software (RadiAnt DICOM Viewer, version 2024.1; Medixant, Poznań, Poland) (Fig. 1).

Additionally, they had a professional hygiene session one week before surgery.

All surgeries were performed by a single specialist in Oral Surgery with extensive clinical experience in the extraction of impacted mandibular third molars and in piezosurgery techniques (A.A.). Each surgery was scheduled at least four weeks apart to avoid confounding factors when recording outcome parameters.

Each patient assumed a single dose of antibiotic prophylaxis one hour before surgery, consisting in 2 g of amoxicillin or 600 mg of clindamycin in case of amoxicillin allergy. Immediately before surgery, patients rinsed for one minute with a 0.20% chlorhexidine gluconate solution (Curasept, Curaden HealthCare, Italy). Combined block anesthesia of inferior alveolar, lingual nerve, and buccal nerve was obtained with 2% mepivacaine and 1:100,000 epinephrine (Optocain, Molteni Dental, Italy). The surgery started ten minutes after the anesthesia administration, with the incision of a triangular mucoperiosteal flap with a #15c scalpel blade.

### Piezoelectric surgery protocol

In the TG, dissection of the mucoperiosteal flap was performed using a piezoelectric device (PIEZOSURGERY® white, Mectron s.p.a., Genova, Italy) with the PR1 insert (Mectron s.p.a.) in *'cancellous'* mode. Once bone exposure was achieved, a MA-HM3M pericoronal osteotomy was performed with the SLO-H insert (Mectron s.p.a.) in *'cortical'* mode up to the cementum-enamel junction. Following this, the EX1 insert (Mectron s.p.a.) was used to deepen the osteotomy in accordance with the root’s angulation.

**Fig. 2:**
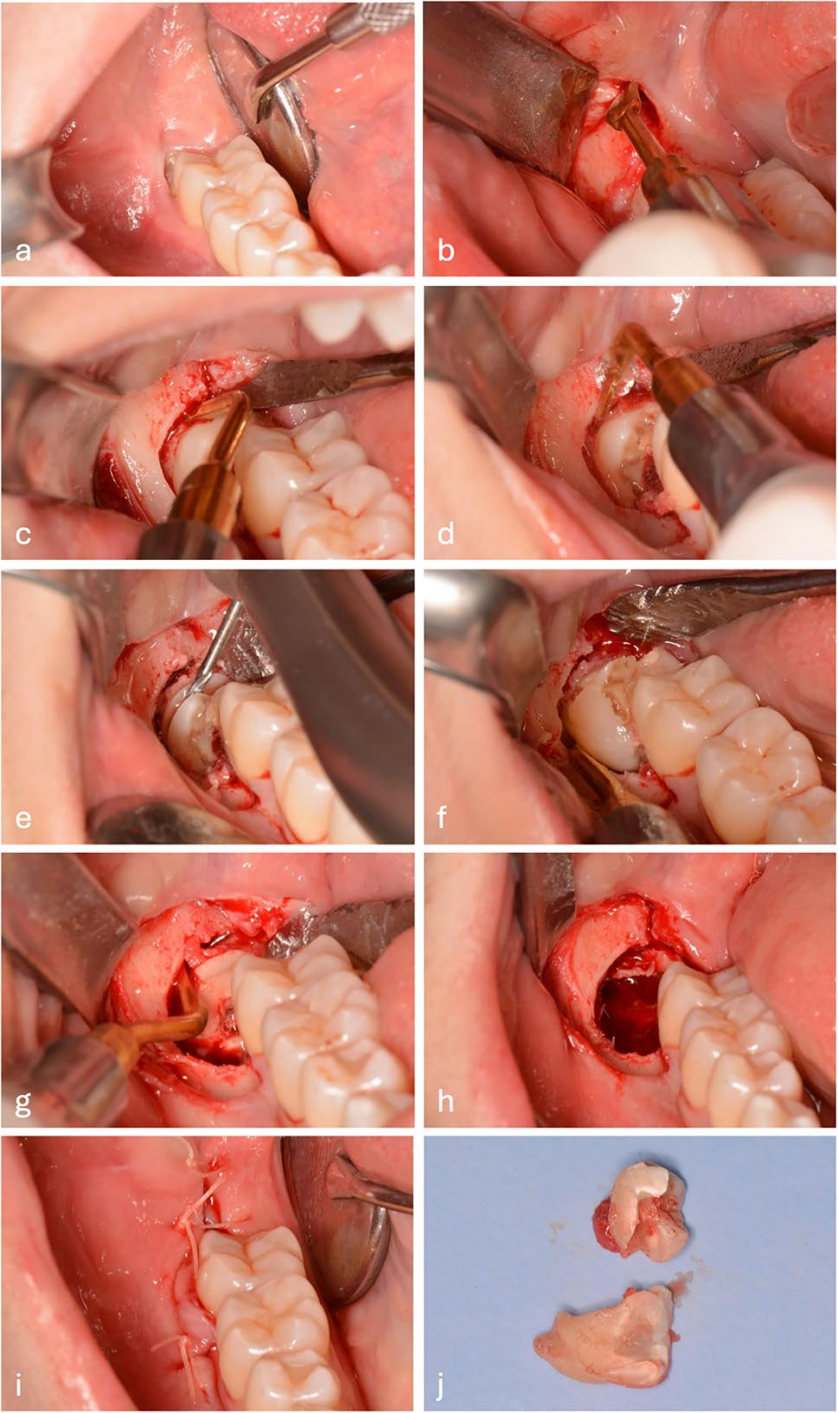
Step-by-step piezosurgery workflow for the removal of a mesio-angulated/horizontally impacted mandibular third molar (MA-HM3M, 48). **a** Preoperative intraoral view showing a fully impacted mandibular third molar (48) in a mesio-angulated/horizontal position; **b** full-thickness mucoperiosteal flap design and elevation performed using the PR1 insert; **c** pericoronal osteotomy extended to the cementum-enamel junction using the SLO-H insert to expose the crown; **d** further deepening of the osteotomy along the axis of the tooth using the EX1 insert; **e** crown sectioning performed with a high-speed handpiece and a fissure bur; **f** removal of the coronal fragment and initial luxation of the tooth using the EXL1 insert; **g** Progressive luxation and atraumatic extraction of the root complex with the EXL3 insert; **h** Intraoperative inspection and refinement of the surgical site following tooth removal; **i** tension-free closure of the surgical flap by suturing; **j** extracted mandibular third molar (48)

For each MA-HM3M, crown sectioning and, in case of multi-rooted tooth, root sectioning was performed using a high-speed handpiece equipped with a fissure bur (Komet Dental, Lemgo, Germany). Subsequently, tooth fragments were luxated using piezoelectric inserts EXL1 and EXL3 (Mectron s.p.a.), also known as piezolevers, in *'cortical'* mode. To correctly perform tooth luxation according to the manufacturer's instructions, intermittent force was applied to the handpiece, positioning the piezolevers between the bone and tooth fragments in alignment with the axis of the tooth (Fig. 2).

All procedures involving piezosurgery and rotary instruments were performed under continuous irrigation with cold saline solution; specifically, during piezoelectric luxation maneuvers, the irrigation setting was maintained at a power level of 5–6. After MA-HM3M removal, the wound was examined and irrigated with 0.9% sodium chloride solution. The flap was repositioned and sutured with a 3–0 resorbable suture (Vicryl® Ethicon, Somerville, NJ, USA).

### Traditional surgery protocol

**Fig. 3:**
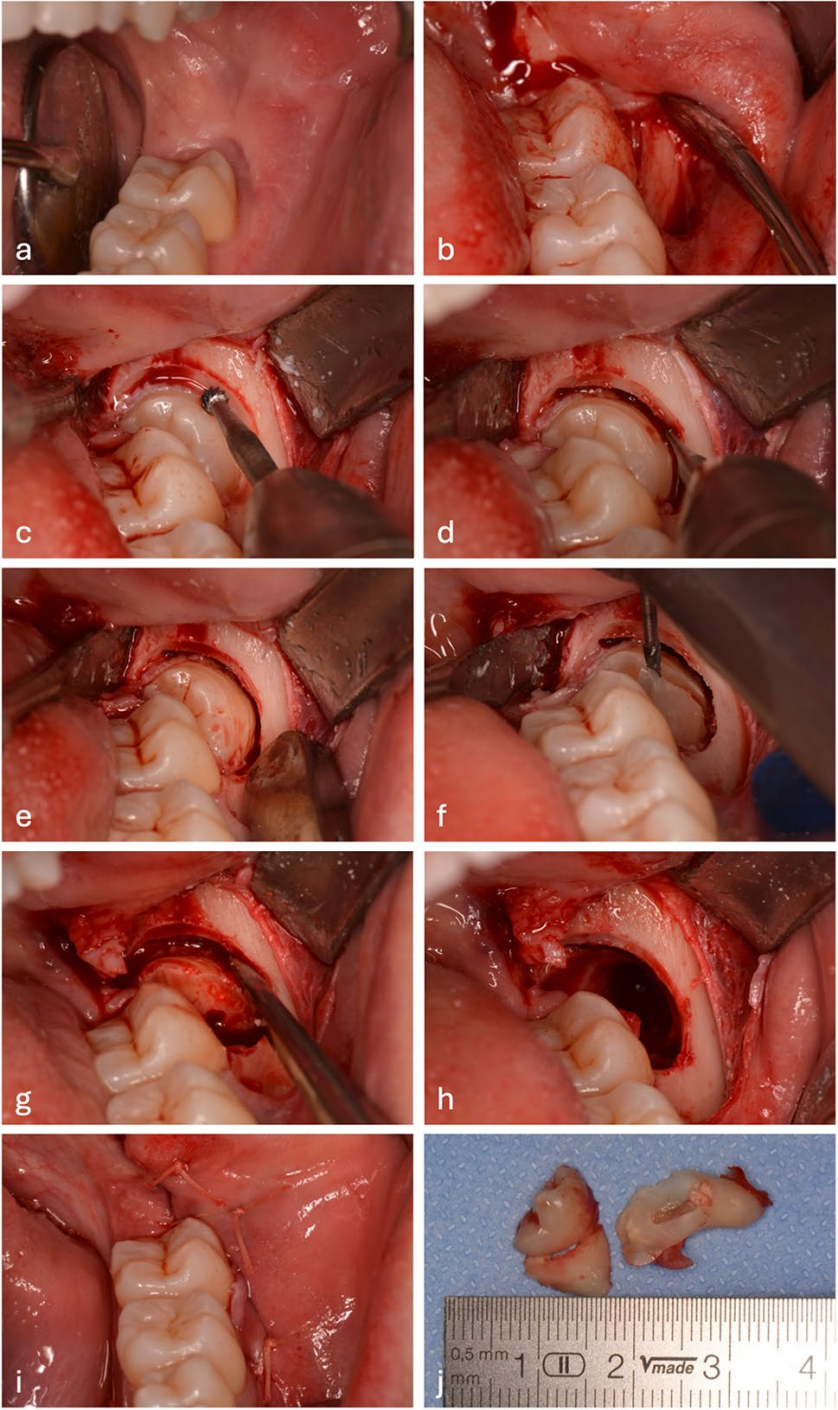
Step-by-step traditional surgical workflow for the removal of a mesio-angulated/horizontally impacted mandibular third molar (MA-HM3M, 38). **a** Preoperative intraoral view showing a fully impacted mandibular third molar (38) in a mesio-angulated/horizontal position; **b** design and elevation of a full-thickness mucoperiosteal flap using a manual Molt #9 periosteal dissector; **c** pericoronal osteotomy extended to the cementum-enamel junction using a straight, low-speed handpiece with a round bur; **d** further deepening of the osteotomy along the long axis of the tooth using a straight, low-speed handpiece with a smaller round bur; **e** complete exposure of the impacted tooth following osteotomy procedures; **f** odontotomy performed with a high-speed handpiece and fissure bur; **g** removal of the coronal fragment and luxation of the tooth using a straight manual elevator; **h** intraoperative inspection and refinement of the surgical site; **i** tension-free closure of the surgical flap by suturing; **j** extracted tooth (38)

In the CG, after mucoperiosteal flap design, flap elevation was performed using a Molt #9 dissector. Osteotomy was carried out using a straight, low-speed handpiece with multiblade round bur (Komet Dental) to expose the MA-HM3M crown and to deepen the osteotomy following the axis of the tooth. Tooth sectioning was performed as above with a high-speed handpiece and a fissure bur (Komet Dental) under continuous saline irrigation. The dislocation and extraction maneuvers of the tooth fragments were performed manually using manual levers (Fig. 3).

Subsequently, the surgical site was examined and washed with 0.9% sodium chloride solution; the surgical flap was repositioned and a 3–0 resorbable suture was applied (Vicryl® Ethicon, Somerville, NJ, USA).

For both surgical protocols, any intra-operative complications were documented at the end of the procedures. Each patient enrolled in the study received the same post-operative protocol: paracetamol 1 g, to be taken up to three times a day as needed after surgery; rinsing with 0.2% chlorhexidine mouthwash for at least 1 min twice a day for one week, starting the day after surgery. The follow-up visits were scheduled two (T1) and seven (T2) days after surgery. Any analgesic intake or adverse event occurred in the first post-operative week was recorded.

### Measurements of outcomes

The primary outcome was facial swelling, quantified through 3D facial scans.

**Fig. 4:**
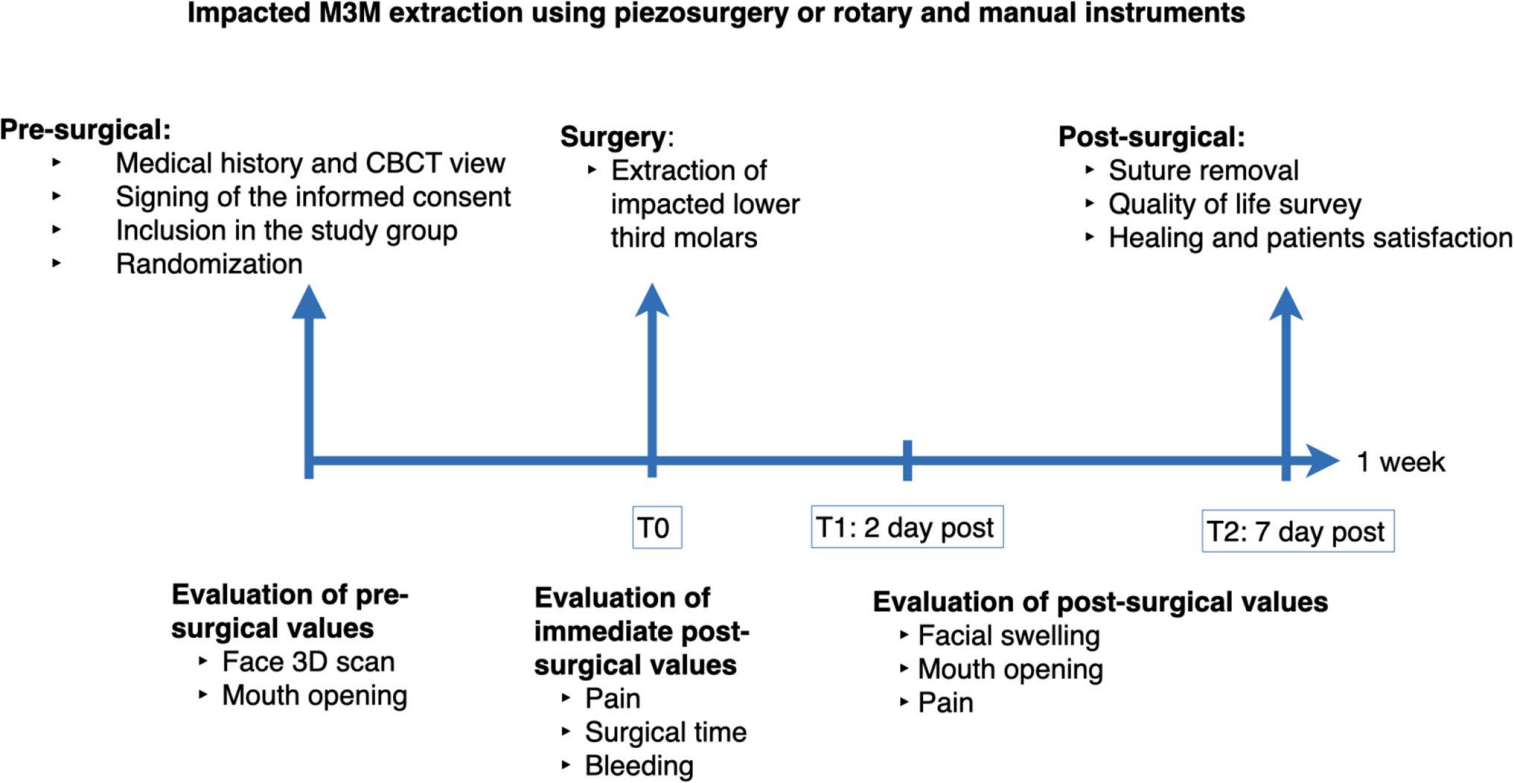
Study workflow and outcome assessment protocol

Secondary outcomes included postsurgical pain, maximum mouth opening, postoperative bleeding, surgery time, complication or adverse event after surgery, analgesic intake, postoperative patients’ quality of life and healing and patients’ satisfaction after surgery (Fig. 4).

#### Facial swelling assessment and data processing method

**Fig. 5:**
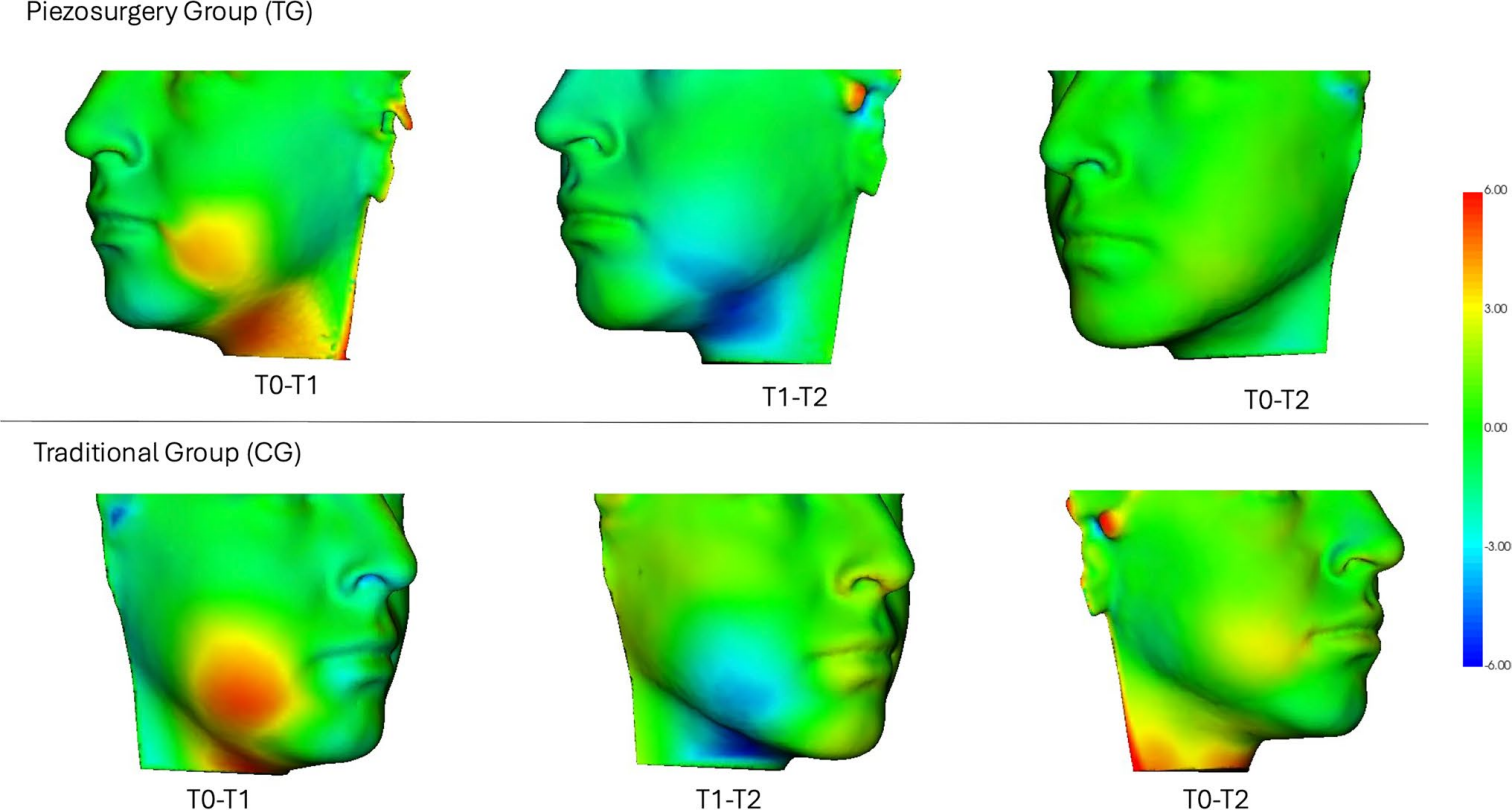
Qualitative analysis of postoperative facial swelling using color map visualization in the piezoelectric (TG) and traditional (CG) groups at three time points. At T0-T1 (2 days after surgery), the CG shows more pronounced swelling, as indicated by the marked red-yellow areas, compared to the test group. From T1 to T2 (day 2 to day 7), the blue areas indicate a reduction in edema. At T0-T2 (7 days after surgery), the TG shows predominantly green areas, indicating the almost complete resolution of facial swelling

To evaluate facial swelling, patients underwent a 3D facial scan three times for each MA-HM3M surgery: immediately before the procedure (T0), at 2 days (T1) and 7 days (T2) postoperatively (Fig. 5).

3D facial images were acquired using the FaceScan MT-4000® (Marathon Italia Srl, Italy), which employs a hybrid structured-light and multi-camera photogrammetry system. All scans were performed following a standardized protocol: patients were seated in a stationary position, facing forward, with the mandible in intercuspal position, lips closed and relaxed. The scanner was fixed in position, and patients were instructed to rotate and tilt their head to allow capture of the entire facial geometry. The scanning result is a 3D triangular mesh of the patient's face, incorporating vertex colors, that can be exported as a.obj,.stl or.ply file.

Digital analysis was performed with the open-source software 3D Slicer (3D Slicer®, The Slicer Community, Open), which incorporates automated tools to apply a defined workflow: 1) data anonymization; 2) orientation; 3) surface registration; 4) qualitative comparisons; 5) linear measurements; 6) volumetric quantification [35].

Each set of facial scans was imported as STL files, generating digital face models (visualization toolkit, vtk files). T1 and T2 facial scans underwent automated surface registration on the T0 scan, which had been previously automatically oriented on the corresponding CBCT, using a standardized method with alignment along anatomical reference planes [36, 37]. The automated tool “*Model-to-Model-Distance*” allowed the superimposition of the facial models in pairs as follow: T0-T1, to evaluate facial swelling two days after surgery; T1-T2, to evaluate any changes in facial edema between 2 and 7 days after surgery; and T0-T2, to evaluate presence of swelling one week postoperatively. To highlight the specific localization of facial edema and to compare differential swelling at different time-points, a colormap was obtained for each superimposed pair using the “*ShapePopulationViewer*” module. Qualitative analysis was conducted by delineating the region of interest (ROI) as the area undergoing tissue change on the colormap. Automated quantification of soft tissue swelling was performed in the identified ROI and involved two main steps: 1) calculation of the mean surface distance differences (in millimeters, mm) on the three paired models using the “*Mesh Statistic*” plugin and 2) quantification of volume differences (in cubic millimeters, mm^3^) between pairs of models using the “*Mesh Volume Comparison*” module.

#### Surgical time recording

Surgical time was recorded for each MA-HM3M removal starting from the flap incision to the end of the tooth extraction, excluding suture procedures.

#### Postoperative pain evaluation

Pain intensity was analyzed using a Visual Analog Scale (VAS) with score from “0 – No pain” to “10 – Worst pain”. Postoperative pain was recorded 4 h after surgery (T0) and at the follow-up appointments (T1 and T2).

#### Assessment of postoperative bleeding

Postoperative bleeding was evaluated after applying local pressure with gauze for 10 min following surgery. Bleeding from the extraction site was then assessed and documented using the following score sheet:

- Score 0: No clinically appreciable bleeding after gauze compression.
- Score 1: Clinically appreciable bleeding after gauze compression; replaced with new gauze and reassessed after an additional 5 min of compression.
- Score 2: Appreciable bleeding after 15 min of compression; requires compressive gauze soaked with tranexamic acid (Ugurol; Rottapharm s.r.l., Monza, Milan, Italy) for 10 min.
- Score 3: Bleeding not controlled by compression and hemostatic drugs; requires the placement of a hemostatic agent such as Tabotamp® (Ethicon™ Biosurgery, Johnson & Johnson MEDICAL GmbH, Norderstedt, Germany) in the surgical alveolus, followed by re-suturing and packing with another sterile compressive gauze; reassessment after 10 min.

#### Mouth opening evaluation

Mouth opening was evaluated immediately before any surgical procedure (T0) using a calibrated steel ruler, referencing the maximum interincisal distance between the marginal portion of upper and lower incisors. At each follow-up visit (T1 and T2), the maximum mouth opening was registered.

#### Postoperative patients’ quality of life

The PoSSe scale, which evaluates criteria such as eating, speech, sensation, appearance, pain, sickness, and interference with daily activities, was used 7 days after surgery (T2) to assess patients' quality of life during the first postoperative week [38]. The score for each item, as well as the total score, was recorded.

#### Healing and patients’ satisfaction analysis

Patients’ healing satisfaction was assessed 7 days after surgery (T2) using a quantitative VAS scale (from 0 – dissatisfied to 10 – completely satisfied). Moreover, 30 days after completing both surgical protocols, patients were asked to indicate their preference for one of the two surgical procedures.

### Statistical analysis

Statistical analysis was conducted using R software (version 4.3.1; http://www.r-project.org). The sample size was calculated given an effect size of 0.5. With a power of 85% and a type I error of 0.05, 44 patients would have been needed. The Kolmogorov–Smirnov test was applied to assess the normality of data distributions. Descriptive statistics included absolute and relative frequencies for categorical variables, means and standard deviations (SD) for continuous variables with normal distributions, and medians with interquartile ranges (IQR) for asymmetrical distributions. For the bivariate analysis, a two-tailed Student's t-test was used for normally distributed data, while the Wilcoxon and Mann–Whitney U tests were applied to compare asymmetrical distributions between the two groups (test vs. control). A linear regression model was used to examine the relationship between post-operative swelling and secondary outcomes within each group. The significance threshold was set at α = 0.05.

## Results

**Fig. 6:**
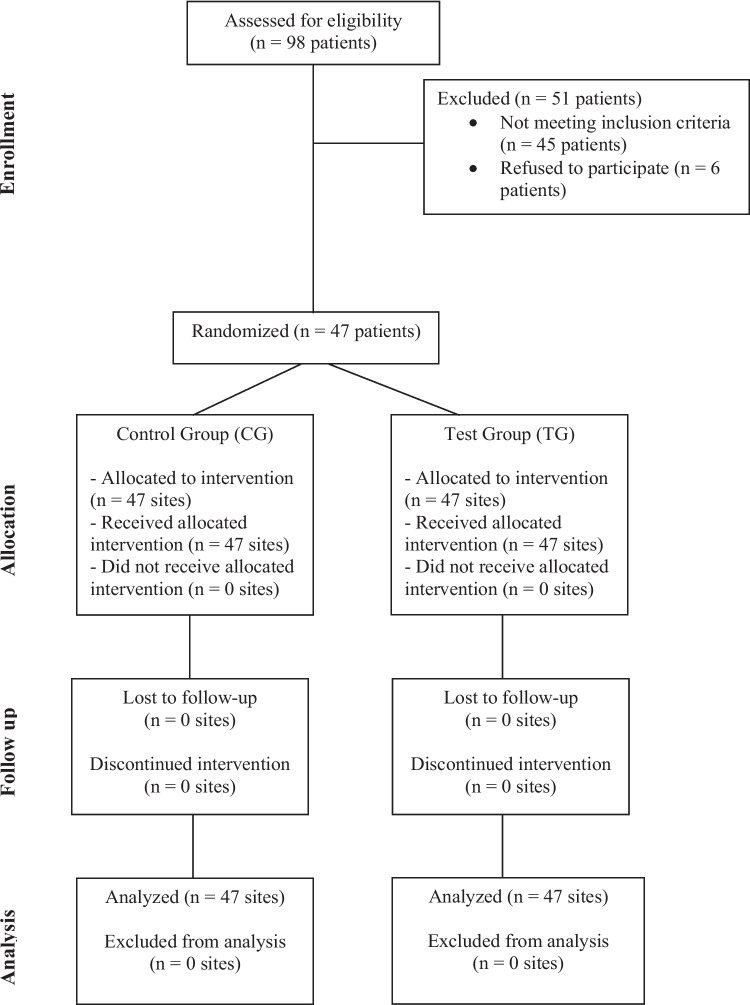
CONSORT diagram illustrating that, out of the 98 eligible patients, 47 patients were included and the sites (right and left) were randomly allocated in two groups. No patient was lost during follow-up procedures

A total of 98 patients were initially screened; of these, 51 were excluded (45 did not meet the inclusion criteria and 6 declined to participate in the study). The remaining 47 patients were included in the study and randomly allocated to TG or CG, resulting in 47 surgical sites per group. No patients were lost to follow-up, and all 47 participants were included in the final analysis (Fig. 6).

Demographic data are reported in Table 1. The study population consisted of 26 females and 21 males, with a mean age of 25 years (range: 18–32 years; SD: 3.7). Overall, 94 M3M surgeries were performed, as each of the 47 enrolled patients underwent both surgical protocols. There were no significant differences in M3M inclination between the groups according to Winter classification (horizontal impaction: 23 in the CG and 21 in the TG; mesioangulated impaction: 24 in the CG and 26 in the TG). No statistically significant differences were observed between the TG and CG in terms of root morphology distribution; the proportion of single-rooted and multi-rooted MA-HM3Ms was comparable between groups.

**Table 1:** Descriptive statistics of the study sample

Variable	Total	Test group (TG)	Control group (CG)	*p*-value
Patients, *n* (%)	47 (100)	–	–	–
MA-HM3M, *n* (%)	94 (100)	47 (50)	47 (50)	–
Sex (female), *n* (%)	26 (55)	–	–	–
Age (years), mean (SD)	25 (3.7)	–	–	–
Smoking status				
– Non-smokers, *n* (%)	23 (49)	–	–	–
– Light smokers, *n* (%)	24 (51)	–	–	–
Impaction pattern				
– Mesio-angulated, *n* (%)	50 (53)	26 (55)	24 (51)	n.s
– Horizontal, *n* (%)	44 (47)	21 (45)	23 (49)	n.s
Root morphology				
– Single-rooted, *n* (%)	32 (34)	15 (31.9)	17 (36.2)	n.s
– Multi-rooted, *n* (%)	62 (66)	32 (68.1)	30 (63.8%)	n.s
IAN contact (CBCT)				
– Yes, *n* (%)	57 (60)	30 (63.8)	27 (57.4)	n.s
– No, *n* (%)	37 (40)	17 (36.2)	20 (42.6)	n.s

Legend. *MA-HM3M* mesio-angulated and horizontally impacted mandibular third molar, *IAN* Inferior alveolar nerve, *n.s.* not significant

A summary of the main outcomes is reported in Table 2.

**Table 2:** Bivariate comparative analysis of the post-operative outcomes in the two study groups (Test group – TG; Control Group – CG)

Outcome variable	Test group (TG)	Control group (CG)	*p*-value
*Swelling_T0-T1*	4865.7 [942.4, 28,005.5]	6563.7 [1054.4, 21980]	0.01*
*Swelling_T0-T2*	1624 [−1166.10, 55,499.60]	2586.50 [−246.00, 14520]	0.26
*Surgical_Time*	21.43 [14.39, 36.04] min	16.55 [9.39, 35.06] min	0.001*
*Postop_Bleeding_Score*	0.55 (0.6)	1 (0.9)	0.037*
*NO_Analgesic_T0*	31 (66.0)	15 (31.9)	0.02*
*NO_Analgesic_T2*	39 (83.0)	29 (61.7)	0.037*
*Pain_T0*	3.00 [1.00, 7.00]	5.00 [0.00, 9.00]	< 0,001*
*Pain_T1*	2.00 [0.00, 9.00]	4.00 [0.00, 8.00]	< 0,001*
*Pain_T2*	0.00 [0.00, 7.00]	1.00 [0.00, 5.00]	0.017*
*Mouth_Opening_T1*	38.00 [21.50, 48.00] mm	36.00 [21.50, 46.00] mm	0.016*
*Mouth_Opening_T2*	41.3 (4.7) mm	39.6 (4.7)	> 0.05
*PoSSe_scale_T2*	16.8 (6.9)	21.6 (5.9)	0.001*
*Healing_Satisfaction_T2*	9.00 [5.0, 10.0]	8.00 [2.0, 10.0]	0.039*
*Patients’ surgical preference*	29 (47)	18 (47)	> 0.05

Legend. *T0* immediately before surgery, *T1* two days after surgery, *T2* seven days after surgery.

P-values refer to between-group comparisons at the same time point

^*^Significant difference (*p*-value < 0.05)

The surgery time showed a statistically significant difference between the groups, being shorter in the CG (16.55 [9.39, 35.06] minutes) compared to the TG (21.43 [14.39, 36.04] minutes; *p* < 0.001).

No differences in postoperative infections or nerve damage were observed during the follow-up period: three cases of alveolitis occurred in the CG and two in the TG. All cases of alveolitis resolved without sequelae within 30 days after surgery. Only three cases of temporary paresthesia were recorded, all in the CG; these completely resolved during follow-up, with full recovery of sensitivity within four months.

The CG also experienced higher levels of postoperative bleeding (*p* = 0.037). Analgesic use was more frequent in the CG at both T0 and T2 (*p* < 0.05).

Both groups experienced increased facial swelling after surgery compared to their initial clinical conditions, with a decrease in swelling from T1 to T2. The volumetric swelling from T0 to T1 was significantly lower in the TG group (4865.7 [942.4, 28005.5]) compared to the CG group (6563.7 [1054.4, 21980]) (*p* = 0.01). Although the TG group showed lower swelling values one week after surgery, there were no significant differences between the groups (TG: 1624.00 [−1166.10, 55499.60]; CG: 2586.50 [−246.00, 14520.00]; *p* = 0.26).

Four hours after surgery, there were different levels of local pain recorded (TG: 3.00 [1.00, 7.00]; CG: 5.00 [0.00, 9.00]; *p* < 0.001). During follow-up clinical evaluations, pain levels decreased at T1 and T2 for both groups. According to the primary predictor variable, TG showed significantly lower pain levels compared to CG at both T1 (*p* < 0.001) and T2 (*p* = 0.017).

Before surgery, all patients had mouth opening values of 42.8 mm (SD 4.7 mm). At T1, the maximum mouth opening was significantly reduced, with higher values in the TG group (38.00 [21.50, 48.00] mm) compared to the CG group (36.00 [21.50, 46.00] mm) (*p* = 0.016). The inflammatory effect persisted one week after surgery, with no significant differences between the groups (TG: 41.3—SD 4.7 mm; CG: 39.6—SD 4.7 mm) (*p* > 0.05).

Healing satisfaction reported by the patients during recovery period was significantly higher in the TG compared to the CG (*p* = 0.039). Overall, 29 out of 47 patients reported higher satisfaction with the piezoelectric approach, whereas 18 patients preferred the conventional technique. However, this difference did not reach statistical significance (p > 0.05). The PoSSe scale analysis highlighted that the “interference with daily life” was significantly lower in the TG group (16.8—SD 6.9) compared to the CG group (21.6—SD 5.9) (*p* = 0.001). Pain, daily activities, eating, and appearance were the most influential factors (*p* < 0.05).

In both groups, the linear regression model showed a significant direct correlation between post-operative swelling and PoSSe scale (*p* < 0.05). In the TG, longer surgery times were significantly associated with increased pain and bleeding, while in the CG, longer surgery times were linked to post-surgical swelling and bleeding (*p* < 0.05). No significant relationship was found between intra-operative root fracture and surgery time in the TG, whereas a significant correlation was observed in the CG (*p* < 0.05) (Table 3).

**Table 3:** Linear regression model showing the significative correlation among the outcome variables and the secondary variables in the two study groups (Test group – TG; Control Group – CG)

	Estimate	Standard error	*p*-value
Test group—TG			
Swelling T1–T2			
Intercept	0.1	0.28	0.73
Posse Scale Total	−0.05	0.02	0.004*
Pain T0			
Intercept	0.08	0.96	0.9
Surgery Time	0.13	0.04	0.0037*
Bleeding			
Intercept	−1.04	0.36	0.006*
Surgery Time	0.07	0.01	< 0.001*
Surgery Time			
Intercept	23.2	0.83	< 0.001*
Root Fracture	2.61	1.75	> 0.05
Control group—CG			
Swelling T1–T2			
Intercept	−1689.6	1610.4	0.29
Surgery Time	495.3	85.8	< 0.001*
Swelling T0–T1			
Intercept	−0.2	0.39	0.62
Posse Scale Total	0.08	0.02	< 0.001*
Bleeding			
Intercept	−1.09	0.35	0.003*
Surgery Time	0.11	0.02	< 0.001*
Surgery Time			
Intercept	19.1	0.91	< 0.001*
Root Fracture	−3.29	1.62	< 0.001*

Legend. *T0* immediately before surgery, *T1* two days after surgery, *T2* seven days after surgery

^*^Significant difference (*p*-value < 0.05)

## Discussion

Surgical trauma resulting from impacted third molar extraction often induces inflammation, leading to facial swelling, pain, and limited mouth opening [15]. These postoperative sequelae are influenced by surgical- and patient-related factors, including procedure duration, technique, surgeon experience, tissue injury, age, systemic health, and ongoing therapies [13, 39]. To reduce post-surgical discomfort, various therapeutic approaches and minimally invasive surgical protocols have been proposed [20, 21, 29, 40].

The aim of this randomized split-mouth clinical trial was to evaluate the performance of a new piezosurgery protocol compared to the traditional approach for the removal of MA-HM3M, focusing on key post-operative sequelae and patient-centered outcomes.

Although previous studies have examined the advantages of piezosurgery for osteotomy in third molar surgery, this is, to the best of our knowledge, the first randomized split-mouth trial to adopt a completely piezoelectric protocol, except for tooth sectioning, and to compare it directly with the traditional technique under standardized clinical conditions [30].

In contrast to the study by Fontanella et al., which included different types of third molars, the present investigation focused on a more homogeneous patient sample in terms of age and degree of dental impaction [13, 31, 39]. Only mesio-angulated or horizontally impacted mandibular third molars (MA-HM3Ms) with comparable inclusion features within the same patient were included.

Moreover, to assess the differences in performance between piezoelectric and traditional extraction techniques while minimizing potential biases related to sample heterogeneity, a split-mouth design was adopted.

This study focused on the main postoperative sequelae following impacted MA-HM3M surgery [15]. 3D facial swelling evaluation showed post-surgical edema at T1, which decreased by T2, with the TG showing significantly less swelling at T1 and no significant difference at T2. Interestingly, the CG showed a more pronounced reduction in edema from T1 to T2.

Unlike the findings of Fontanella et al., who reported no significant differences in facial swelling, our results may be attributed to the stricter inclusion criteria, the different piezoelectric surgical approach and the use of automatic 3D swelling assessment, which has been shown to be more accurate and reliable than traditional 2D methods [31, 35, 41–44].

Our results are in line with previous clinical trials that compared piezosurgery and rotary instrumentation in M3M extraction, reinforcing the evidence for a positive association between the piezoelectric approach and reduced postoperative facial swelling [45–47]. As emphasized in recent meta-analyses, these results are likely attributable to its selective, minimally invasive and micrometric cutting action, which reduces local trauma and soft tissue injury, thereby attenuating the inflammatory response [48].

This reduced inflammatory reaction may also contribute to the lower postoperative pain levels observed in the TG. Pain peaked at 4 h after surgery in both groups but remained significantly lower in the TG at all postoperative timepoints. These findings align with results from previous split-mouth RCTs, which reported lower pain levels following piezoelectric surgery [49, 50]. In contrast, Fontanella et al. reported no significant differences in pain scores, which may be attributed to sample heterogeneity and differences in surgical protocols compared with the present study [31].

Postoperative trismus assessment revealed a reduction in maximum mouth opening in both groups; however, this limitation was significantly greater in the CG compared to the TG at two days from surgery. Consistent with our findings, Piersanti et al., in a split-mouth RCT involving an age-homogeneous sample, reported significantly greater trismus with the traditional technique compared to the piezoelectric approach, specifically at the two-day postoperative follow-up [51]. Analogous favorable results with piezosurgery were also documented by Barone et al. and Sortino et al., who employed a mouth opening assessment method similar to that used in the present study [46, 47].

Our results are consistent with recent systematic reviews and meta-analyses showing that the piezosurgery approach significantly reduces postoperative complications following third molar extraction [30, 52]. This benefit could be mainly attributable to its micrometric and minimally traumatic osteotomy, which limits the extent of bone involvement [26, 53, 54].

Reduced biological tissue damage is a hallmark of osteotomy performed with piezosurgery; its selective action on mineralized tissues minimizes the inflammatory insult, often resulting in less facial swelling, pain, and trismus [49–51]. Conversely, manual and high-speed rotary instruments may lead to overheating and unintended soft tissue injury during osteotomy and dissection procedures [55, 56].

The reduced inflammatory response and related discomfort in the TG are further supported by postoperative analgesic consumption registered in our study, which was significantly higher in the CG both immediately after surgery and throughout the first week of follow-up [52, 57, 58].

Postoperative bleeding was significantly lower in the TG compared to the CG, and this finding could be attributed to the cavitation effect and the micrometric cutting action, which make piezoelectric technique less traumatic than conventional methods [59–61].

Operative time was significantly longer in the TG; this data has been consistently reported in the literature and represents the main drawback of piezosurgery [28, 30]. However, several authors have highlighted that operative time decreases with surgeons’ experience [31]. Furthermore, it should be considered the operative time spent in locking and unlocking the 4 different inserts on the piezoelectric handpiece. This time-consuming procedure is not present in the CG.

Prolonged operative time is generally considered a potential contributing factor to postoperative morbidity, as longer surgical procedures may increase tissue manipulation, flap retraction, intraoperative stress, and local inflammatory response. Consequently, extended surgery duration could theoretically lead to greater postoperative swelling, pain, and trismus. Interestingly, despite the significantly longer operative time observed in the TG, patients treated with the piezoelectric protocol still showed more favorable postoperative outcomes in most evaluated parameters. This finding suggests that the reduced mechanical and thermal trauma associated with piezoelectric surgery may partially compensate for, or even outweigh, the potential negative effects related to increased surgical duration [26–29].

Notably, our regression analysis revealed that the increased operative time was associated with higher postoperative bleeding in both groups, possibly reflecting the need for more extensive surgical maneuvers [62]. Interestingly, the relationship between surgical time and postoperative sequelae differed between groups: in the TG, longer surgeries were associated with higher postoperative pain, while in the CG, they correlated with greater swelling. These findings may reflect variability in the postoperative course between the two groups and could be partially influenced by differences in surgical dynamics and tissue handling [62–66].

No correlation was observed between operative time and root or apex fractures in the TG, whereas in the CG, longer procedures were significantly associated with a higher incidence of root fractures. These findings suggest that the piezoelectric protocol provides greater control and precision, particularly in challenging dental extractions. The use of dedicated piezoelectric tips for tooth luxation and root removal offers a mechanical advantage, allowing for minimally invasive yet effective luxation through selective bone removal [31]. This approach facilitates the creation of adequate space around the tooth, allowing deeper insert advancement and more efficient application of luxation forces [31]. Beyond clinical outcomes, patient-reported outcome measures (PROMs) provide critical insights for a comprehensive evaluation of the two techniques [66].

Lower levels of discomfort in eating procedure, less pain, reduced swelling, and fewer limitations in daily activities were reported in TG compared to CG. These findings are consistent with other clinical studies, confirming that the subjective postoperative experience is significantly more favorable with the piezoelectric approach [50, 51].

Although 62% of patients expressed a preference for the piezoelectric technique, the difference did not reach statistical significance. However, when evaluating perceived healing of the surgical site, a significant preference was observed for the piezoelectric protocol. This may reflect a more favorable biological healing process, as supported by histological data from Horton et al., which showed more organized tissue architecture and less cellular degeneration in sites treated with piezosurgery [67].

Despite an increasing number of studies has compared these two surgical approaches, heterogeneity in sample selection and outcome assessment has hindered the establishment of clear consensus.

The strength of the present study lies in its rigorous design, characterized by strict inclusion criteria (MA-HM3Ms only), use of piezosurgery not limited to osteotomy, standardized surgical procedures, and objective 3D assessment of postoperative swelling.

When interpreting the present findings, the use of antibiotic prophylaxis should also be considered. In accordance with the recommendations reported by Milic et al., a single preoperative antibiotic dose was administered to all participants. This regimen was applied uniformly across both study groups to ensure standardized perioperative management, while acknowledging that the role of antibiotic prophylaxis in third molar surgery remains a matter of ongoing debate in the literature [68]. Current evidence suggests that a single preoperative antibiotic dose may reduce the risk of postoperative infectious complications; however, its overall clinical benefit appears modest and should be carefully weighed against the principles of antimicrobial stewardship [69–71]. Importantly, because the same antibiotic protocol was consistently administered to all patients, any potential effect was equally distributed between groups and is therefore unlikely to have influenced the comparative outcomes of the present study. The main limitations of the study include its focus exclusively on a specific M3M type, which may affect the generalizability of the findings, and the four-week interval between the two procedures, which could potentially influence patient perception and subjective outcome reporting.

## Conclusions

The results of this split-mouth randomized controlled trial suggest that this piezoelectric surgical approach for the extraction of MA-HM3Ms may effectively reduce postoperative inflammatory sequelae compared with the conventional technique based on manual levers and rotary instruments. These improvements were associated with better patient-reported postoperative quality of life during the early healing period.

Although the piezoelectric protocol required longer operative times, this aspect did not appear to negatively influence postoperative morbidity. Moreover, despite higher costs and the need for dedicated equipment and software, 3D facial scanning allowed an objective and reproducible assessment of postoperative edema, potentially reducing the operator-dependent variability and measurement bias commonly associated with conventional manual or analogic evaluation methods.

Further multicenter split-mouth randomized controlled trials with larger and more heterogeneous samples are needed to better clarify the benefits and potential short-term complications associated with this surgical approach, including different types of mandibular third molars and operators with different levels of surgical experience. In addition, despite ethical and practical limitations, future histological investigations may help to further elucidate the biological tissue response associated with piezoelectric and conventional surgical techniques.

## Data Availability

No datasets were generated or analysed during the current study.

## Declarations

### Artificial intelligence declaration

During the preparation of this work, the authors used ChatGPT Plus in order to linguistic sparring. After using this tool, the authors reviewed and edited the content as needed and take full responsibility for the content of the publication.
